# Punctuality in Infant Feeding

**Published:** 1902-05-31

**Authors:** 


### Punctuality in Infant Feeding.

We lately drew attention to a case in the King's Bench Division of the High Court of Justice in which it had been decided on appeal that a purchaser of new milk might be defrauded, in that he might not get an article of the nature, substance, and quality demanded, even though the milk supplied to him could be shown to have come direct from the cow without having undergone adulteration or sophistication of any kind. In the particular case in question the cow's excuse for producing the fraudulent milk which led to all this litigation was that she had not been milked for some 16 hours, and that by this delay in the time of milking milk becomes so deteriorated as no longer to be what the Judge described as "ordinary milk." What we wish now to point out is that human milk may be deteriorated in the same way, namely, by allowing an abnormal length of time to intervene between the "milkings," and that although the baby cannot hale its mother before the King's Bench for not supplying it with an article of the nature and quality demanded, it can punish her in other ways, notably by causing many a restless night and by giving an infinite amount of trouble in its bringing up. It is, of course, perfectly well known that the food, the work, and the regime of the mother affect the milk which she produces, but it is perhaps not quite so fully recognised as it ought to be that mere want of punctuality in the times of giving the breast, may have such a deteriorating influence as to render the milk unsuitable as a food, and that this unpunctuality is the cause of a good deal of the difficulty which some women experience in nursing their infants.

There is a strong feeling among many women of experience that it does not do to "mix the milk," that is to say, that if the bottle is to be given at all it had better be given entirely, to the complete exclusion of the natural food. And mothers are able to bring a good deal of practical experience to support their contention. But what is this experience? Often that of women who, either from the demands of work or the calls of pleasure, use the bottle as a means of getting away from their baby for a considerable number of hours at a stretch. It is an experience derived from the use not of mixed but of deteriorated milk —milk deteriorated by delay in the milking. If a mother is to feed her infant every three hours she clearly cannot go to the factory, nor can she go much into society. Thus it is that many women who maintain that they are nursing their own children, do in fact give several "bottle meals" a day, not with the object of supplementing an inefficient supply, but with the definite purpose of putting off the time of nursing. But would any dairyman who wanted his cows to go on producing good milk allow them to be milked at just such hours as might be convenient to the milkmaids? Certainly not.

Yet a woman thinks nothing of going to work all day and then giving her infant three or four feeds in the next 12 hours, and many a lady who takes pride in the fact that she is nursing her baby herself endeavours by aid of an occasional "bottle" to take her full share of many pleasures which must of necessity take her away from her nursery for hours together. Indeed, some are only limited in their absences by the discomfort to themselves and the injury to their dresses that arise if they "go too long." No decent self-respecting cow would stand such treatment, and we need hardly be surprised that milk drawn under such circumstances should be found not to be of the quality demanded by the infant. Hence, no doubt, some of the prejudice against bringing up a child partly on bottle and partly on breast. Among working people, and indeed among mothers of all degrees who are so situated as to have to look after their own children at night, it is a common practice to continue giving baby the breast at night long after it has taken to ordinary food in the daytime. This is done for several reasons. Firstly there is the ever-present dread of beginning the process of maternity over again—a constant nightmare to most married women—then there is the maternal gratification of giving pleasure to her babe, and lastly there is the not unnatural dislike to getting up in the night to prepare the infant's food. But not only is this nocturnal feeding extremely exhausting to the mother, but there is much reason to believe that milk drawn in such an irregular manner, and after such a long interval, provides but a poor sort of nutriment for the child. In the words of the Act, it is not "of the nature, substance, and quality demanded."

What, then, we would urge in regard to the natural, in contradistinction to the artificial feeding of infants, is that it is not enough that the mother should suckle her own child, nor is it enough that for the sake of producing good milk she should keep herself in good health, taking plenty of nourishing food, keeping the natural functions of the body in good order, living a good deal in the open air, and getting a proper amount of exercise, but that, in addition to all this, she should take at least as much care as a dairyman would do that the milk shall not be spoiled by irregularity in the hours of milking.

